# Characterisation of Children's Head Motion for Magnetic Resonance Imaging With and Without General Anaesthesia

**DOI:** 10.3389/fradi.2021.789632

**Published:** 2021-12-03

**Authors:** Hannah Eichhorn, Andreea-Veronica Vascan, Martin Nørgaard, Andreas H. Ellegaard, Jakob M. Slipsager, Sune Høgild Keller, Lisbeth Marner, Melanie Ganz

**Affiliations:** ^1^Neurobiology Research Unit, Copenhagen University Hospital, Rigshospitalet, Copenhagen, Denmark; ^2^Niels Bohr Institute, University of Copenhagen, Copenhagen, Denmark; ^3^Department of Computer Science, University of Copenhagen, Copenhagen, Denmark; ^4^Center for Reproducible Neuroscience, Department of Psychology, Stanford University, Stanford, CA, United States; ^5^TracInnovations, Ballerup, Denmark; ^6^Department of Applied Mathematics and Computer Science, Technical University of Denmark, Lyngby, Denmark; ^7^Department of Clinical Physiology, Nuclear Medicine & PET, Copenhagen University Hospital Rigshospitalet, Copenhagen, Denmark; ^8^Department of Clinical Physiology and Nuclear Medicine, Copenhagen University Hospital Bispebjerg, Copenhagen, Denmark

**Keywords:** magnetic resonance imaging, motion patterns, motion artefact, pediatric radiology, anaesthesia, brain

## Abstract

Head motion is one of the major reasons for artefacts in Magnetic Resonance Imaging (MRI), which is especially challenging for children who are often intimidated by the dimensions of the MR scanner. In order to optimise the MRI acquisition for children in the clinical setting, insights into children's motion patterns are essential. In this work, we analyse motion data from 61 paediatric patients. We compare structural MRI data of children imaged with and without general anaesthesia (GA), all scanned using the same hybrid PET/MR scanner. We analyse several metrics of motion based on the displacement relative to a reference, decompose the transformation matrix into translation and rotation, as well as investigate whether different regions in the brain are affected differently by the children's motion. Head motion for children without GA was significantly higher, with a median of the mean displacements of 2.19 ± 0.93 mm (median ± standard deviation) during 41.7±7.5 min scans; however, even anaesthetised children showed residual head motion (mean displacement of 1.12±0.35 mm). For both patient groups translation along the z-axis (along the scanner bore) was significantly larger in absolute terms (GA / no GA: 0.87±0.29/0.92 ± 0.49 mm) compared to the other directions. Considering directionality, both patient groups were moving in negative z-direction and thus, out of the scanner. The awake children additionally showed significantly more nodding rotation (0.33±0.20°). In future studies as well as in the clinical setting, these predominant types of motion need to be taken into consideration to limit artefacts and reduce re-scans due to poor image quality.

## 1. Introduction

For Magnetic Resonance Imaging (MRI), artefacts are most frequently caused by patient head motion due to long acquisition times of typically 30–60 min (1). These artefacts manifest as ghosting, blurring or signal variations, thus reducing overall image quality and resulting in unsuccessful diagnoses (2). Andre et al. (3) determined the percentage of at least partly repeated MRI examinations to approximately 20%, leading to an estimated increased cost of 115,000 US dollars per scanner per year in the US due to motion.

Children tend to move more in the MR scanner than adults since they are intrinsically more reluctant to lie still and are more negatively influenced by the large size of the scanner, the narrow bore and the loud noises during image acquisition (4). So far, motion artefacts for children between 4 and 10 years are mostly reduced by sedation or general anaesthesia (GA), with different GA rates reported for different age ranges: 61–100% for children between 4 and 6 years (5), 80% for children aged 1-6 years (6) and 40% for children aged 7–12 years (6). However, the need for an anaesthetist to administer the drug and monitor the child, increases the patient's waiting times, as well as the costs of the examination. Slipsager et al. (7) calculated the additional cost for using GA in MRI examinations to 319,000 US dollars per scanner per year in Denmark. Furthermore, there are concerns about adverse events like airway obstruction or oxygen desaturation of GA in young children (1, 4, 8, 9).

Currently, different approaches for preventing motion artefacts without general anaesthesia or sedation are under research. These include strategies for preparing the children with story books and mock scanners before they undergo MRI and distracting them visually and acoustically during the examination (4, 5, 10–12). Another approach is to apply motion correction during or after image acquisition, for which a variety of methods have been developed and tested. These include prospective motion correction updating the position of the field of view in real time dependent on motion estimates (13–15), as well as several retrospective techniques using mathematical properties of the Fourier Transform, Compressed Sensing or Machine Learning (16–19).

For both strategies—preparation and motion correction—information about the children's motion patterns in the MR scanner is essential. In the case of motion correction algorithms, additional information is needed for tailoring them toward children specific movements. For the preparation strategy as well as in the clinical setting, information about predominant motion patterns is critical for preventing those types of motion. Churchill et al. (20) analysed head motion of adults during fMRI using retrospective image-based motion correction. Overall, they found low motion estimates with only two cases of estimates above 1mm or 1° for the whole brain. The largest standard deviation of the displacement relative to a reference image was observed for pitch movement (nodding). Afacan et al. (1) investigated children's head motion and its impact on image quality. Their analysis showed a correlation of mean displacement and motion free time to image quality, but no statistically significant correlation of maximum displacement. Additionally, they did not find a significant correlation between the analysed motion metrics and age. Together, these results suggest that head motion is a challenge, not only for young and uncooperative children.

The aim of this work is to analyse motion patterns of children in order to draw possible conclusions about predominant movement habits and thus, enable optimisation of motion correction methods for specific types of motion. For that, we also investigate how head motion translates into motion of different parts of the brain and whether different regions in the brain are affected more or less severely by the children's motion. The analyses will be performed for children imaged with and without GA.

## 2. Materials and Methods

### 2.1. Patient Population

**Figure 1:**
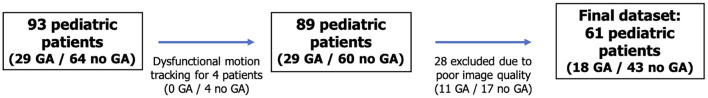
Schematic overview of exclusion process.

Within this study, MR and motion data from 93 paediatric patients with brain tumors acquired in a previous study were analysed (21, 22). As visualised in Figure 1, four of those data sets were excluded due to dysfunctional motion tracking. Twenty eight additional data sets were excluded due to failed image processing with FreeSurfer (23)—because of poor scan quality of the MPRAGE scan (ringing or blurring), large tumors or removal of large parts of the brain. Examples of excluded scans as well as a comparison of motion between excluded and included patients are provided in the Supplementary Materials 1, 2. Consequently, the final dataset consists of 61 patients aged between 21 months and 19 years, out of which 18 were scanned with GA (propofol and sevoflurane) and 43 were scanned without. Additional demographic information is provided in Table 1. Please note the larger age range of anaesthetised children, which is due to clinical considerations.

**Table 1:** Demographic information for patient population.

	**GA**	**No GA**	**All**
Number of patients (sex)	18 (10 male)	43 (28 male)	61 (38 male)
Mean age ± std	5.94 ± 2.75	12.65 ± 3.04	10.67 ±4.25
Age range	1–13	7–19	1–19

The previous study, in which our data was acquired, was approved by the Danish National Committee on Health Research Ethics (approval H-6-2014-095) and was registered at clinicaltrials.gov (NCT03402425). Written informed consent was obtained from all patients / parents of the patients.

### 2.2. MRI Acquisition and Region Segmentation

Clinical MR scans were performed using the mMR Biograph hybrid PET/MRI scanner (*Siemens Healthineers, Erlangen, Germany*) between April 2015 and January 2019, using a PET/MR child brain tumour protocol. Before the procedure, the included children were kept in warm and friendly surroundings with their parents/caregivers, while preparing for scanning. Ear plugs were used during the scan to shield from scanner noise. A microphone ensured contact with the scanner technicians for children without GA. In case of anxiety, the parents would sit by the child during the scanning procedure.

**Figure 2:**
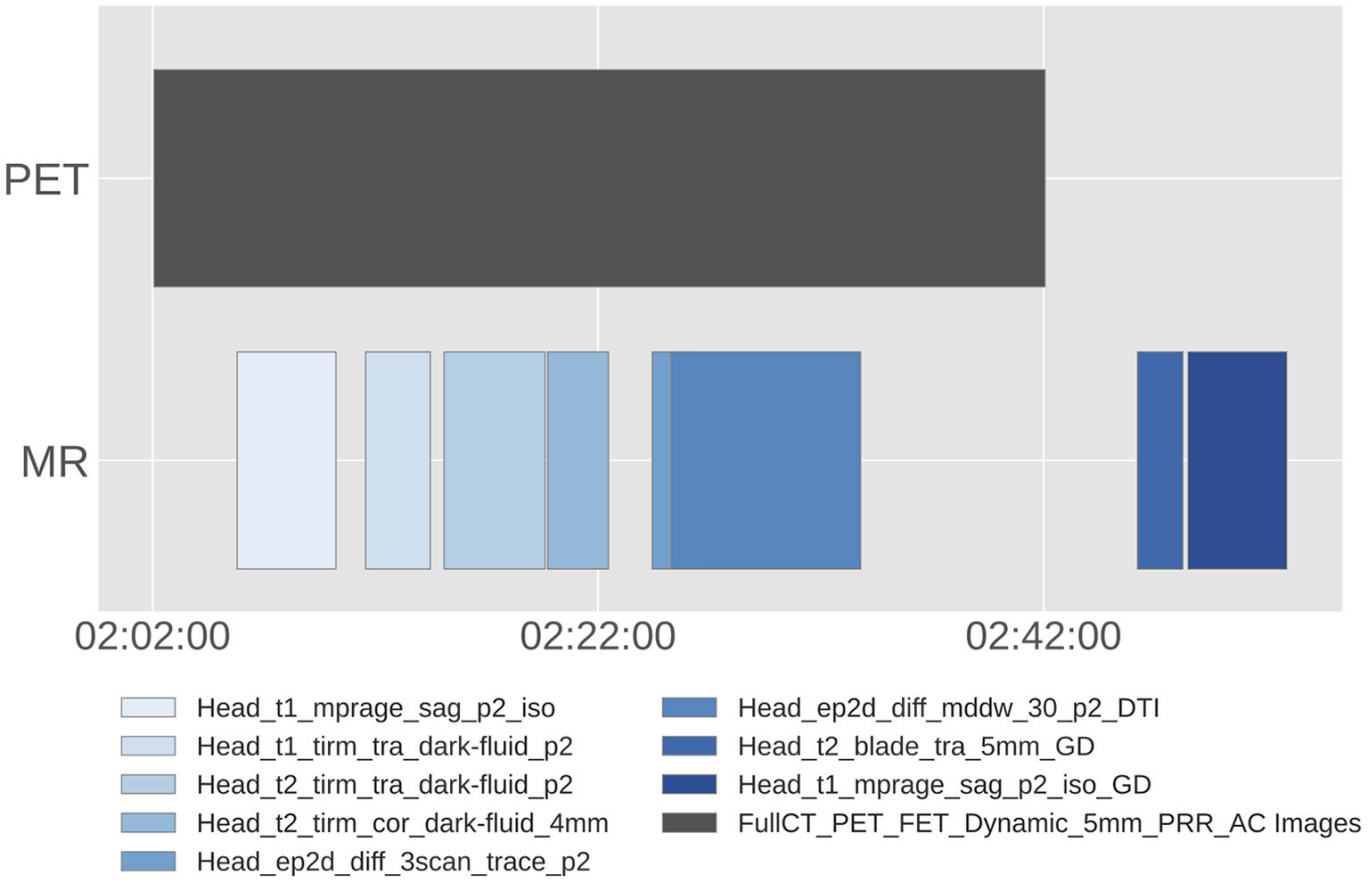
Example diagram of PET and MR scans for an individual patient. The specific MR sequences used, as well as their acquisition order differed from child to child, the base sequences of the protocol are listed in Table 2. Sequences acquired using gadolinium as contrast agent are labelled with “GD.”

The median wall time from the first to the last MR sequence was 41.7±7.5 min. The MRI sequences used differed from child to child, the base sequences of the protocol are listed in Table 2. A diagram for an example scan session is provided in Figure 2. Please notice that the protocol consisted of both MR and PET scans acquired in an interleaved fashion, which is why the median wall time from the first to the last MR sequence was longer than the sum of the scan durations in Table 2. Parameters of these sequences are reported in the Supplementary Material (Supplementary Table 1). The quality of the majority of the 61 scans in the final dataset was assessed as “optimal for clinical use” by radiologists, with only 6.3% of the scans scored “useful for diagnosis, but not optimal,” as previously described by Slipsager et al. (7).

**Table 2:** MR Sequences of the PET/MR child brain tumour protocol.

**Sequence**	**Scan duration [s]**
T_1_-weighted MPRAGE	266
T_1_-weighted STIR	174
T_2_-weighted FLAIR (transversal)	272
T_2_-weighted FLAIR (coronal)	164
T_2_-weighted Blade	122

*Note that the protocol consisted of both MR and PET scans, which is why the median wall time from the first to the last MR sequence was longer than the sum of the scan durations*.

**Figure 3:**
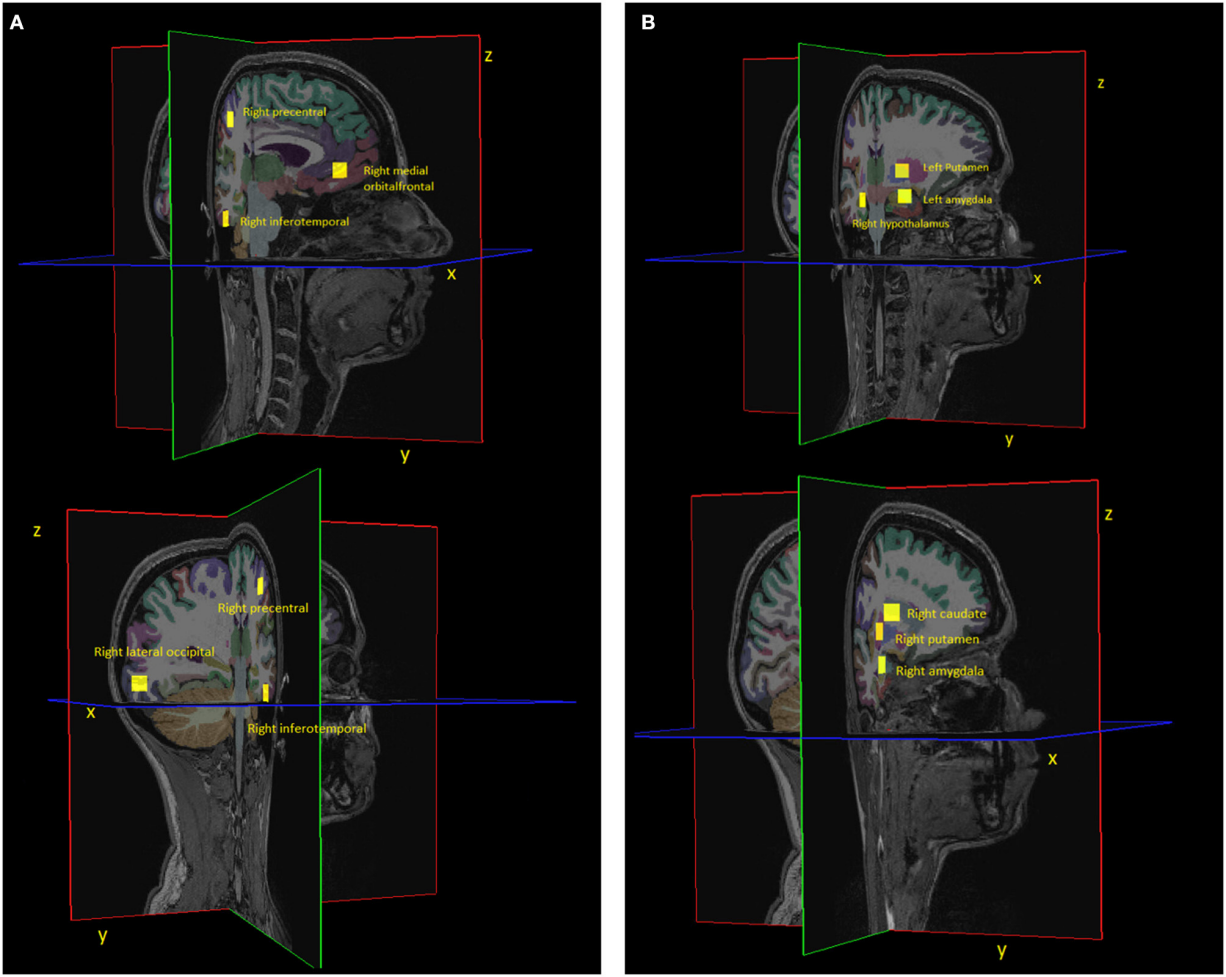
Visualisation of **(A)** 4 cortical and **(B)** 4 subcortical regions together with their centroid (yellow square). Please note that the centroid is a single point, but we visualised it as a square in this figure for better visibility. The center of the square corresponds to the centroid coordinate. Due to viewing purposes, we only show half of the regions that were included in the analysis. The other half consists of the symmetrical regions in the respective other brain hemisphere.

The 3D-encoded, T_1_-weighted MPRAGE scans were processed with FreeSurfer (23) in order to segment 8 cortical and 8 subcortical regions: left and right hippocampus, caudate, amygdala, putamen, lateral occipital, inferior temporal, precentral and medial orbitofrontal regions. The centroids of these regions are visualised in Figure 3.

### 2.3. Motion Tracking

The patient's head motion was estimated with the markerless tracking system Tracoline (*TracInnovations, Ballerup, Denmark*) (24, 25). It transmits non-visible infrared light onto the patient's face and estimates a 3D point cloud surface, the position of which is continuously measured at a frequency of approximately 30 Hz. An example of this point cloud for an adult as well as a schematic visualisation of the point cloud centroid is shown in Supplementary Figure 3 of the Supplementary Material. A rigid-body transformation matrix was determined for each time point by registering the corresponding point cloud to a reference point cloud. Our analysis in the following was only performed on the motion data sampled during acquisition of the MR sequences summarised in Table 2. The motion data measured during dead time as well as during PET scans was disregarded, since we were only interested in the children's motion patterns during MR acquisition and the connected challenges e.g., due to the high noise level. For each motion estimate, the middle time point of the MPRAGE acquisition was chosen as the reference position, since the segmentation of brain regions was estimated from the MPRAGE scan and this time point corresponds to the acquisition of the centre of k-space—the time during which the acquisition is most sensitive to motion. Using a cross-calibration transform, the estimates obtained in the Tracoline coordinate system were moved into the RAS (Right, Anterior, Superior) coordinate system.

### 2.4. Motion Quantification

#### 2.4.1. Motion Metrics

In line with the analysis of Afacan et al. (1), the displacement of the point cloud centroid to the reference position was calculated for all time points as Euclidean distance to the reference position. For each patient, the mean, median and maximum of the displacement was determined. We chose to report both mean and median displacement, since the displacements over time were not normally distributed, which we confirmed with a Kolmogorow-Smirnow test for normality. Additionally, motion-free time was quantified as the percentage of time, where the displacement relative to the reference position was below 2 mm. This threshold was chosen different from Afacan et al. (1), since 2 mm is a standard value for motion thresholding at our institute and in our experience, 2 mm is a realistic and practicable threshold with respect to image quality.

Analogously, in order to compare how rigid-body head motion affects different regions of the brain, the rigid-body transformation matrices for all time point were applied to the regions' centroids (cf. Figure 3). In this way the displacements of the regions' centroids and the corresponding metrics were computed.

#### 2.4.2. Matrix Decomposition

Tracoline provides an estimate of the rigid body transformation matrix at each time point. Analogous to Churchill et al. (20), we decomposed each transformation matrix into translational and rotational components, which reveal the translation (mm) along and rotation (degrees) around the three axes.

### 2.5. Statistical Analysis

Statistical differences in motion metrics as well as in absolute translational and rotational components between groups and across brain regions (*N* = 16) were evaluated using the Mann-Whitney *U*-test. Correction for multiple comparisons across regions was carried out using False-Discovery Rate (26) (FDR), at FDR = 0.05.

### 2.6. Code Availability

The code used to preprocess and create the results used in this manuscript mostly consists of open- source software (FreeSurfer and Python). Code for running the analysis can be found at GitHub: https://github.com/melanieganz/MoCoProject/tree/main/ChildrenHeadMotionMRI.

## 3. Results

### 3.1. Exemplary Motion Curves

**Figure 4:**
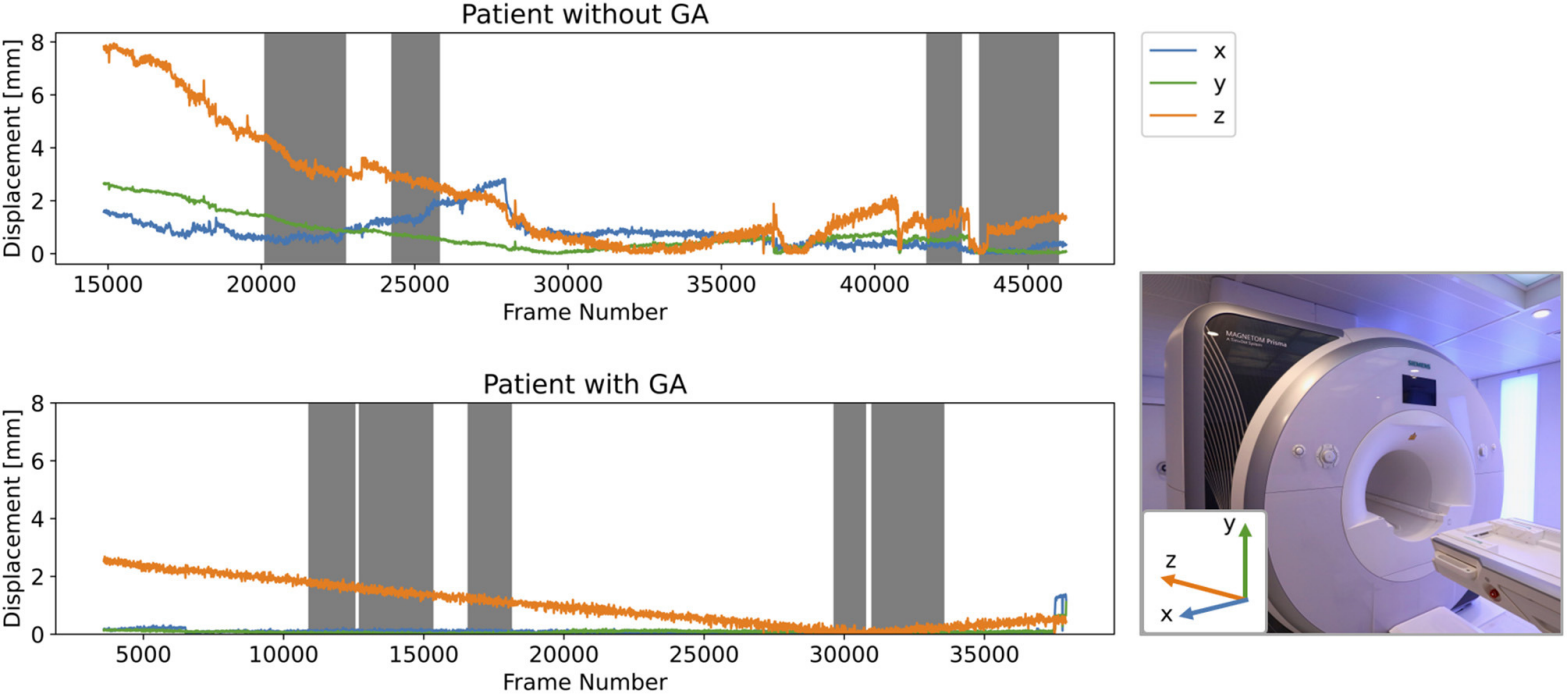
Example of motion curves for children with and without GA. Grey background indicates the times where MR sequences have been acquired. The x-, y-, and z-axes in the RAS coordinate system are visualised relative to the MR scanner in the image inset.

Exemplary motion curves during the entire scan session are shown in Figure 4 for one patient with and one without GA. Curves for other GA patients are similar to the shown example, whereas the curves for awake children show more variation.

### 3.2. Comparison of Motion Metrics

**Figure 5:**
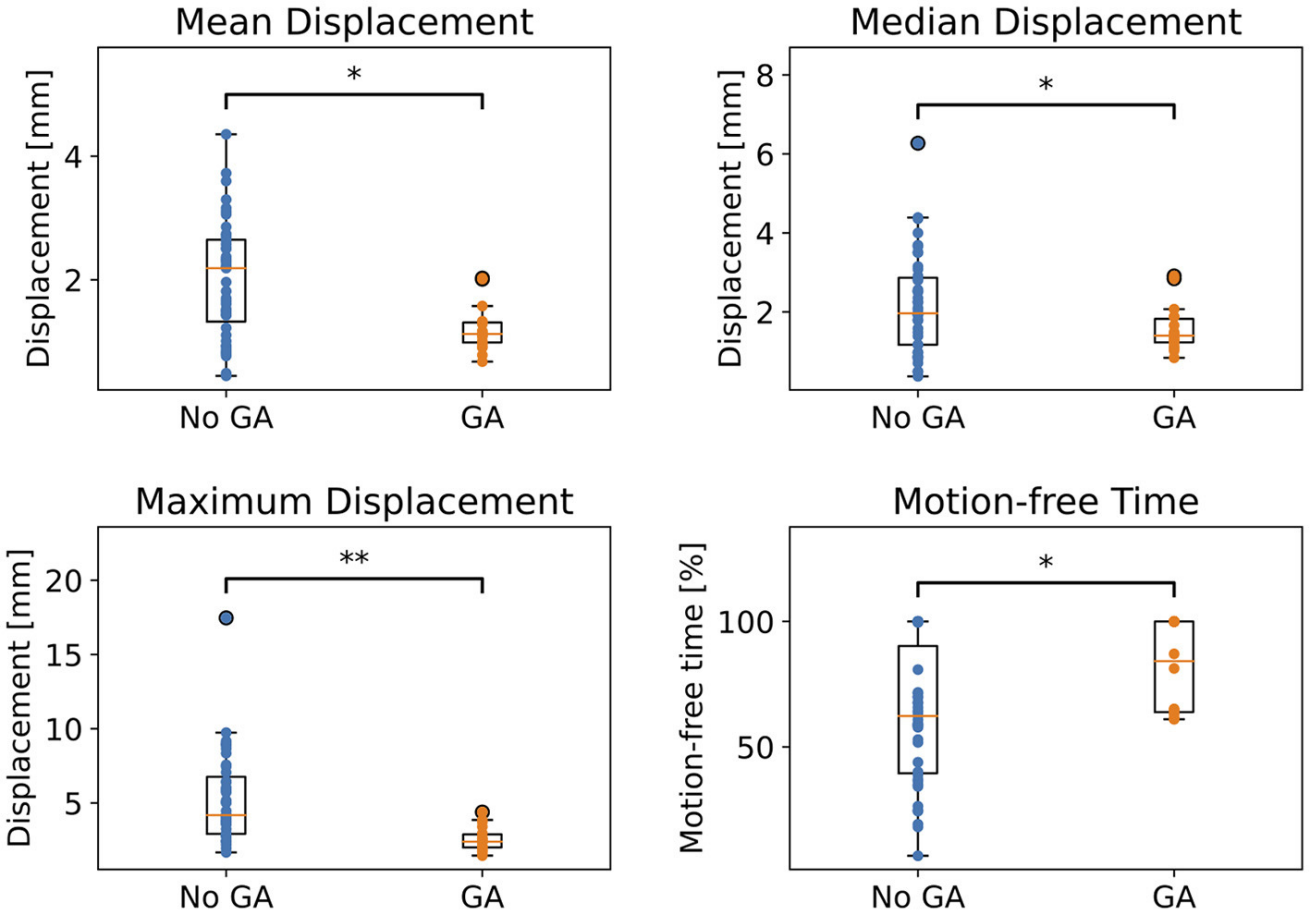
Comparison of motion metrics calculated with point cloud centroid for children with and without GA. Statistical significance after FDR correction is indicated by */** (*p* < 0.05/*p* < 0.001).

Figure 5 compares mean, median and maximum displacement, as well as motion free time for patients with and without GA. All metrics show a statistically significant difference between anaesthetised and awake children. Please note even for children under anaesthesia several metric values, like motion-free times down to only 58%—corresponding to an amount of motion that might affect image quality.

The metrics for the 16 analysed brain regions can be compared in Supplementary Figures 4–7 of the Supplementary Material. Maximum and mean displacement as well as motion free time vary statistically significantly between patients with and without GA for all regions. Median displacements are distributed over a larger range and only show statistical significance for some regions, which could be explained by the fact that median values are not as strongly affected by outliers as mean values. However, the same trend as for the other metrics is still observable.

### 3.3. Decomposition of Motion Into Translation and Rotation

**Figure 6:**
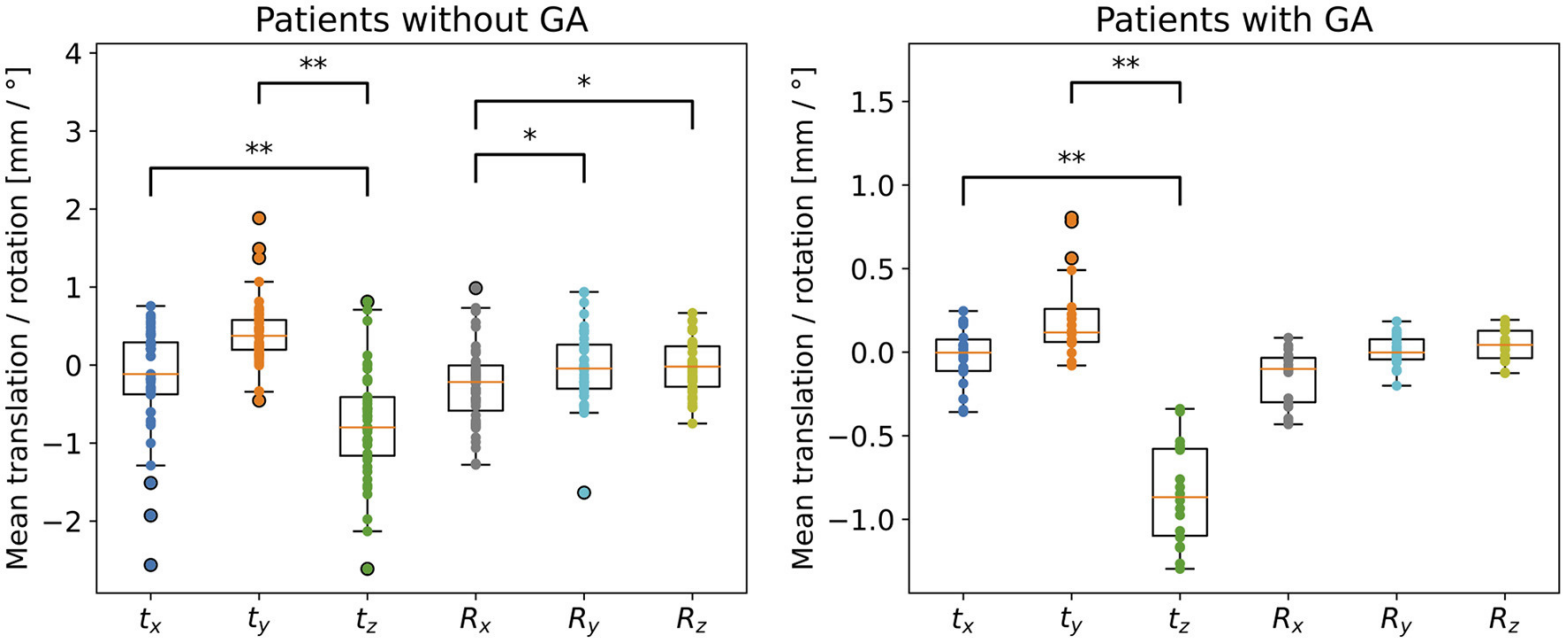
Decomposition of transformation matrices into translational (*t*_*x*_, *t*_*y*_, *t*_*z*_) and rotational components (*R*_*x*_, *R*_*y*_, *R*_*z*_), for children without and with GA. Statistical significance after FDR correction is indicated by */** (*p* < 0.05/*p* < 0.001); please note that significance was tested on absolute translations and rotations in order to detect size differences in motion parameters, whereas mean values of positive and negative components are shown in the graph for visualising the direction of the motion. Notice the different scales for children without and with GA.

We extracted translational and rotational components for both patient groups and compare the components along the different axes within each patient cohort in Figure 6. For both groups, the absolute translational component along the z-axis, which is the axis going into the scanner bore and pointing toward the top of the head, is significantly larger than the absolute values along the x- and y-axis. Regarding directionality of this translation, both patient groups move in the negative z-direction, i.e., slide downwards out of the scanner. For the children without GA, the absolute rotational component around the x-axis is significantly larger than the components around the other axes, whereas for the GA group no significant differences between the rotations are observed. In the RAS system, the x-axis points from right to left.

### 3.4. Motion of Different Brain Regions

**Figure 7:**
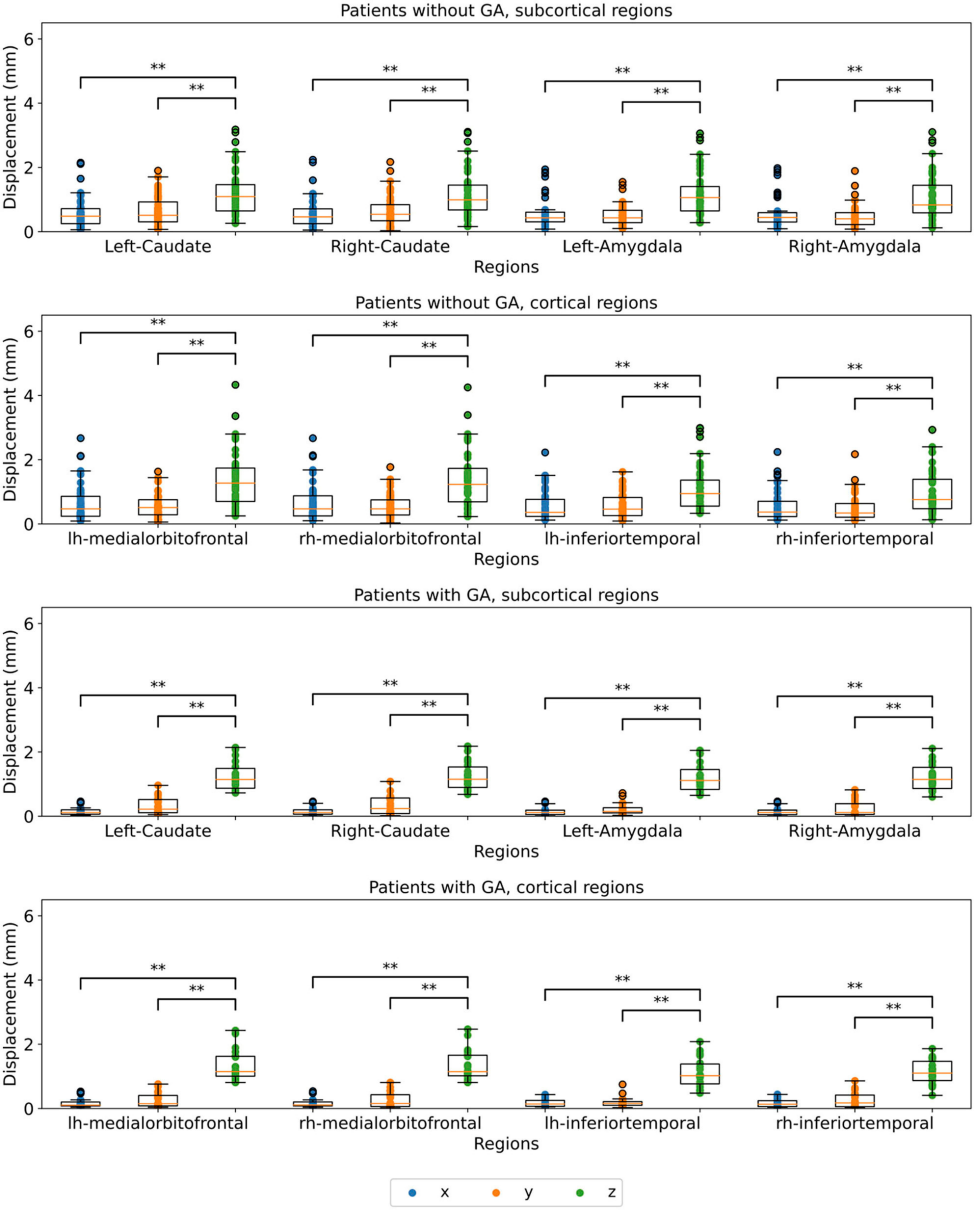
Comparison of median displacement on each axis for 4 cortical and 4 subcortical regions split for the x-, y, and z-axis (blue, orange, and green, respectively). The upper two plots show patients without GA, the lower two patients with GA. Statistical significance after FDR correction is indicated by */** (*p* < 0.05/*p* < 0.001).

To further analyse how the children's rigid-body head motion translates into motion of different brain regions, we calculated the median displacement on the x-, y-, and z-axis for each brain region, respectively. Figure 7 shows the results for 4 cortical and 4 subcortical regions. The results for the remaining 8 regions analysed in this study are available in the Supplementary Material (Supplementary Figure 8). The observed displacements did not seem to differ from one brain region to another based on visual assessment. The magnitude of the motion on the z-axis exceeds that of the other axes for both patient groups, even though the differences are smaller for the group without GA. This difference is statistically significant for both groups and all 16 regions except the comparison of z- and y-axis for the right hemisphere precentral region in the patient group without GA (see Supplementary Figure 8).

## 4. Discussion

We analysed motion data from anaesthetised and awake children during MRI examination using the same MR scanner and a variable set of sequences from the same base protocol for all patients, which to our knowledge has not been reported so far. Please note that the order and specific parameters of the sequences varied from patient to patient, since the data was acquired in a clinical study.

Comparing motion metrics like mean, median, maximum displacement and motion-free time in Figure 5, confirmed our expectations that children without GA moved significantly more than children with GA for all analysed metrics. Nevertheless, residual movement was still observed for anaesthetised children. As described in the Introduction, Afacan et al. (1) investigated the correlation of motion metrics like mean and maximum displacement and motion-free time with image quality based on motion estimates, which were measured with two sensors placed on the patient's forehead. For enabling comparisons with their work, we calculated our metrics for the point cloud centroid, which corresponds to a point close to the middle of the nose bridge, as visualised in Supplementary Figure 3. Even though Afacan et al. did not find significant differences between radiologic evaluation and maximum displacement, we reported maximum movement since it provides valuable information about the severity of motion spikes, especially in the context of motion correction.

Furthermore, in Figure 5, we showed across all metrics that even anaesthetised children moved: with mean displacements of up to 1.9 mm, maximum displacements of up to 4.4 mm and motion-free times down to 58 %. Afacan et al. (1), who analysed the correlation of motion metrics with radiologic evaluation of image quality, reported motion-free time medians of 38%± 21, 74%± 27, 97%± 17 and 99%± 3 for the grades 1, 2, 3, and 4, respectively (4 being the best image quality). Together with our results of motion-free time values down to 58%, this indicates that GA does not guarantee high image quality due to considerable residual motion in several cases. Our different threshold for calculating motion-free times, does not falsify these conclusions, since lowering our threshold from 2 to 0.2 mm as in (1)] would only decrease our percentages of motion free times. Please note that Afacan et al. also used a different motion tracking device, an electromagnetic tracker (27) developed by Robin Medical Inc. (Baltimore, MD). For other motion tracking devices using external fitted tools, like the Polaris Vicra, inaccuracy in the motion estimates has been shown due to dislocation of the tool relative to the head (28).

In order to obtain more insight into which types of movement children perform most commonly during MR examinations, we decomposed the transformation matrices for each time point into translation and rotation. For both patient groups, we compared the components along / around the three axes. In Figure 6, we showed for both patient groups significantly larger absolute translation along the z-axis compared to x- and y-axes. For the children without GA, we additionally observed significantly larger rotations around the x-axis compared to the y- and z-axes. The z-translation, which corresponds to translation along the axis through the scanner bore, can be interpreted as a drift motion, especially for the children with GA. This becomes apparent when looking at the complete motion data throughout the whole scan session e.g., in Figure 4. For most of the GA patients one can observe a continuous increase in the displacement along the z-axis from the beginning until the end of the examination. Since we observed negative z-translation values for both patient groups, this motion corresponds to a gliding downwards out of the scanner and could be explained both by relaxation of the child's neck muscles, as well as compression of the foam padding which the head lies on. The rotational component around the x-axis observed for children without GA corresponds to a nodding motion, which together with a sliding in negative z-direction has previously also been observed as primary motion for adults in the MR scanner (20, 29). For children specifically, this movement can be explained by parents or a screen with a movie being positioned at the end of the scanner bore. Thus, our analysis provides important conclusions for clinical examinations of children, namely that avoiding nodding motion should play a larger role in training children before an examination.

Moreover, we analysed how the children's motion affects different parts of the brain. For this, we chose 8 cortical and 8 subcortical regions of the brain. We calculated the displacement along each axis for the selected regions. We did not observe a difference when comparing the displacements between regions. The region-wise results were also in accordance with the results from the matrix decomposition presented above. For the GA group very little motion was observed on the x- and y-axes. The group without GA showed more motion on the x- and y-axes, but the z-axis was still dominating. The differences were statistically significant for all regions, except the precentral region of the right hemisphere for the patient group without GA. For this region, the median motion was more distributed across y- and z-axes.

Our study is not without limitations. First, it is limited by the unbalanced distribution between the groups with and without GA. A larger GA group might enable higher statistical significance for comparing the motion metrics between both groups. Second, the examined group of children only consisted of patients with brain tumors. Other patient groups are to be expected to show different motion patterns, for instance some diseases, such as epilepsy or cerebral palsy, come along with an increased tendency for motion. Third, no single motion metric used in the analysis is suitable for summarising the whole motion pattern. For instance a short, but severe nodding increases the maximum displacement, even though the child could have potentially remained motionless for the rest of the scan—with little effect to image quality. However, the combination of several motion metrics (mean, median, and maximum displacement, as well as motion-free time) allows a more complete evaluation of the child's motion, since the different metrics pick up different motion patterns. Furthermore, we only analysed motion patterns and metrics across the whole protocol, which enables us to draw conclusions about which motion children are expected to perform during a whole MR examination. However, we cannot draw conclusions about the motion characteristics and with it, the image quality of individual sequences. Another potential source of error is the fact that FreeSurfer is optimised for adult brain anatomy, which could lead to slightly wrong segmentation of the brain regions. However, each segmentation was verified manually. Lastly, we excluded approximately one third of the data set, amongst other things due to poor scan quality impeding a successful segmentation by FreeSurfer. This could lead to excluding scans with large amounts of motion and thus, underestimating the true motion of children in general. However, our comparison of included and excluded scans in Supplementary Figure 2 confirmed that excluded scans did not systematically have larger amounts of motion, apart from two outlier scans (see Supplementary Figure 2).

## 5. Conclusion

Considering the presented results together with the discussed limitations, our study puts some light onto how children move during a (PET/)MR scan, when they are awake as well as anaesthetised. First of all, we showed that even children under GA show an amount of motion on the order of 1–2 mm mean displacement, which can impact MR image quality. This strengthens the need for alternative methods such as motion correction techniques and adequate preparation of the children to avoid motion artefacts in case the motion causes clinically image degrading artefacts. For the anaesthetised children, the clinical evaluation of image quality reported in this study shows no problem with motion artefacts; free motion, however, requires motion correction to avoid GA for more children. In addition, our data indicates that clinical routines as well as training methods under development should be given special attention to prevent nodding motion. Similarly, the higher prevalence of nodding motion and translation along the z-axis should be taken into account when optimising and testing motion correction methods. In order to limit motion artefacts further, the application of MR sequences that are more robust toward these two types of motion regarding slicing and phase encoding direction should be considered.

## Data Availability Statement

The data analysed in this study is subject to the following licenses/restrictions: due to regulations according to the Danish Data Protection Agency, the very sensitive patient data including the MRI sequences are not available. Requests to access these datasets should be directed to Lisbeth Marner, lisbeth.marner@rh.regionh.dk.

## Ethics Statement

The studies involving human participants were reviewed and approved by Danish National Committee on Health Research Ethics. Written informed consent to participate in this study was provided by the participants' legal guardian/next of kin.

## Author Contributions

Data collection was performed by LM, SK, AE, and JS. HE, A-VV, MN, and MG contributed to the design of the work and analysed the data. The first draft of the manuscript was written by HE. All authors interpreted the data, critically reviewed the manuscript, and approved the submitted version.

## Funding

This work was supported by The Danish Childhood Cancer Foundation (2014-34, 2015-48), as well as the Elsass Fonden (18-3-0147), and an unrestricted Grant from the Novo Nordisk and Novozymes Talent Program.

## Conflict of Interest

AE and JS, as well as MG's husband are employees at TracInnovations, Ballerup, Denmark. The remaining authors declare that the research was conducted in the absence of any commercial or financial relationships that could be construed as a potential conflict of interest.

## Publisher's Note

All claims expressed in this article are solely those of the authors and do not necessarily represent those of their affiliated organizations, or those of the publisher, the editors and the reviewers. Any product that may be evaluated in this article, or claim that may be made by its manufacturer, is not guaranteed or endorsed by the publisher.
