# Supplementary material for: Characterisation of Children's Head Motion for Magnetic Resonance Imaging With and Without General Anaesthesia
Source: Front Radiol. 2021 Dec 3;1:789632. doi: 10.3389/fradi.2021.789632 (PMC10365093; doi:10.3389/fradi.2021.789632)
Supplement: Supplementary file 1 [file Data_Sheet_1.PDF]

Supplementary Material

1 SUPPLEMENTARY TABLES AND FIGURES

1.1 Tables

Table S1. Parameters of the MR sequences describing the type of acquisition, flip angle, repetition, echo as well as inversion time and voxel size.

Sequence	Acquisition type	Flip angle [°]	TR/TE/TI [ms]	Voxel size [mm]
T_1-weighted MPRAGE	3D	9	1900/2.52/900	1.00×1.00×1.00
T_1-weighted STIR	2D	150	2000/34/800	0.45×0.45×4.00
T_2-weighted FLAIR (transversal)	2D	150	9000/85/2500	0.69×0.69×4.00
T_2-weighted FLAIR (coronal)	2D	130	9000/95/2500	0.43×0.43×4.00
T_2-weighted Blade	2D	140	4000/118/—	0.72×0.72×5.00

1.2 Figures

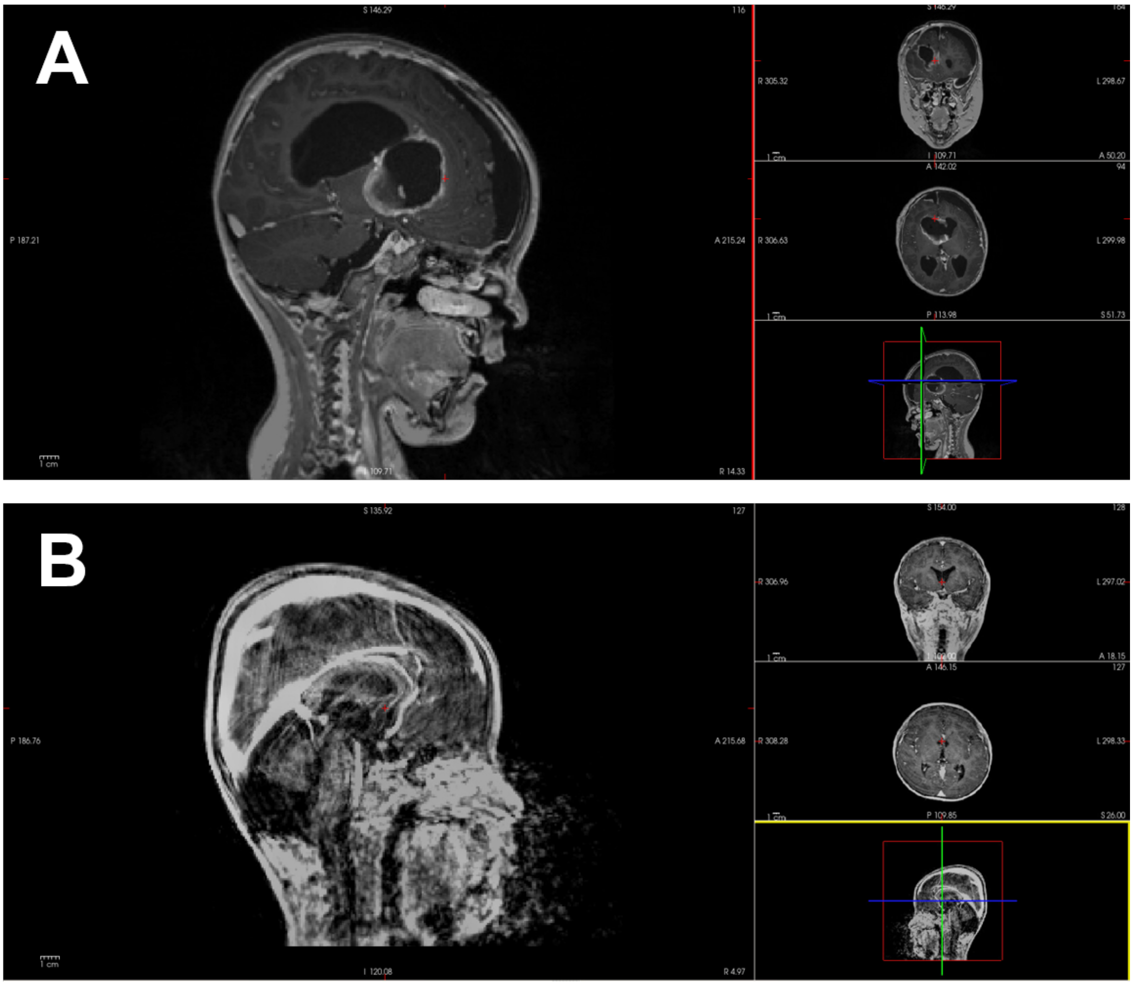

Figure S1. Examples of excluded scans due to (A) large defects and (B) poor scan quality.

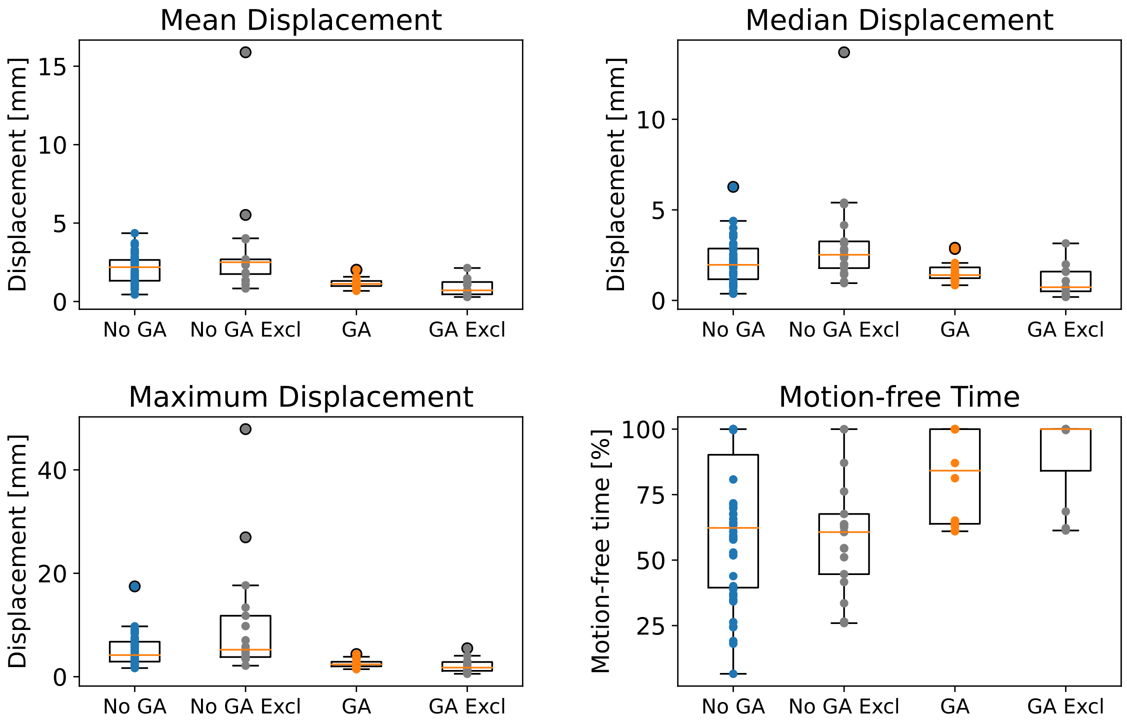

Figure S2. Motion metrics for point centroid of scans excluded due to poor image quality compared to included scans. The analysis of the excluded scans is performed analogously to the analysis shown in Figure 4. Except two outlier scans with significantly larger motion in the group without GA, the excluded patients show similar amount of motion as the included patients.

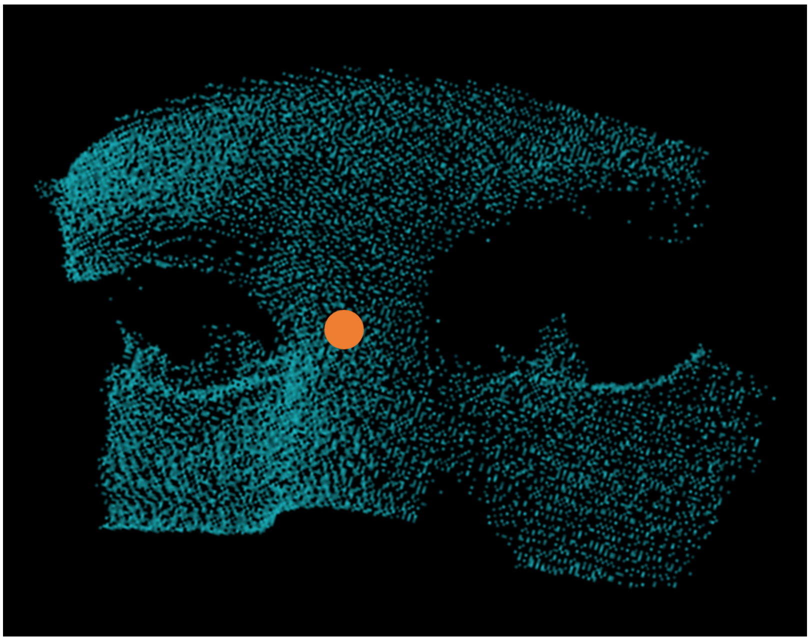

Figure S3. Example of the Tracoline point cloud for a five year old child. The point cloud centroid is schematically visualised by the orange circle. Please note that the field of view varies with head size and curvature of the face.

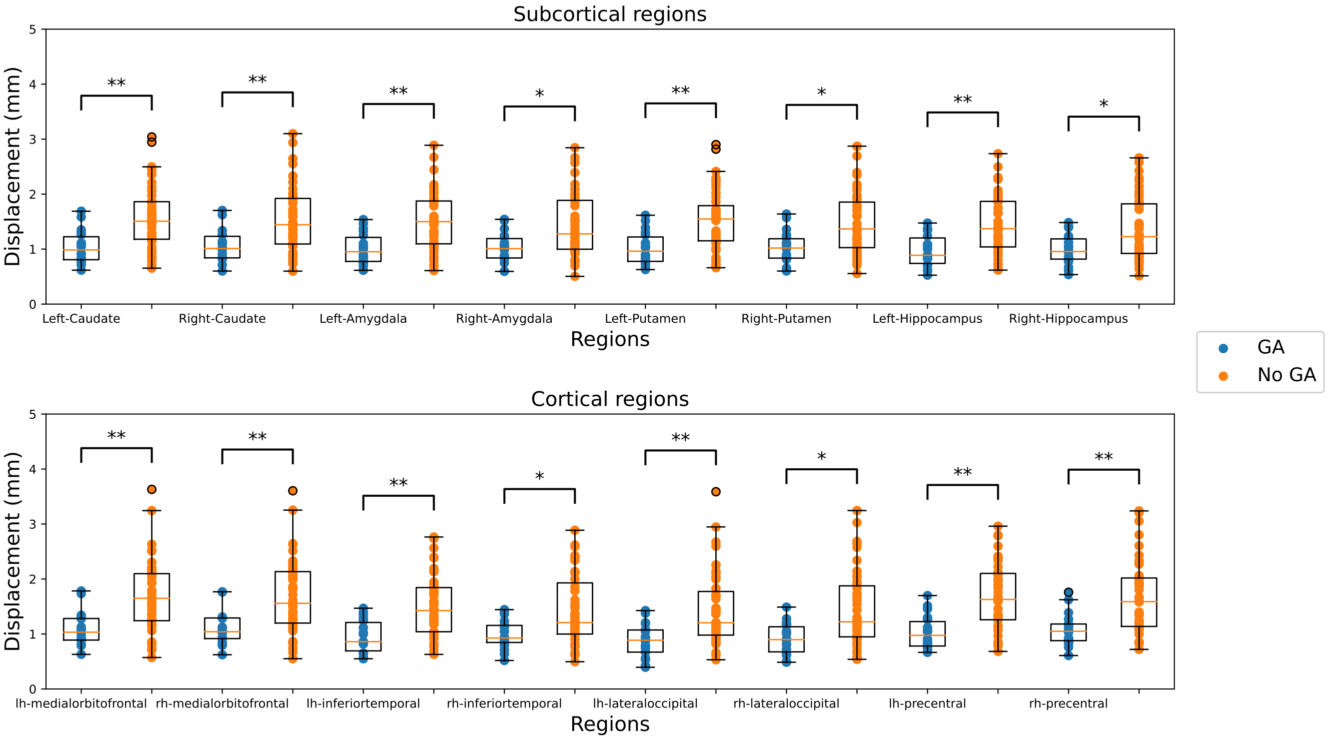

Figure S4. Comparison of mean displacement for all cortical and subcortical regions. Statistical significant differences after Benjamini-Hochberg correction are indicated by */** ($p < 0.05/p < 0.001$).

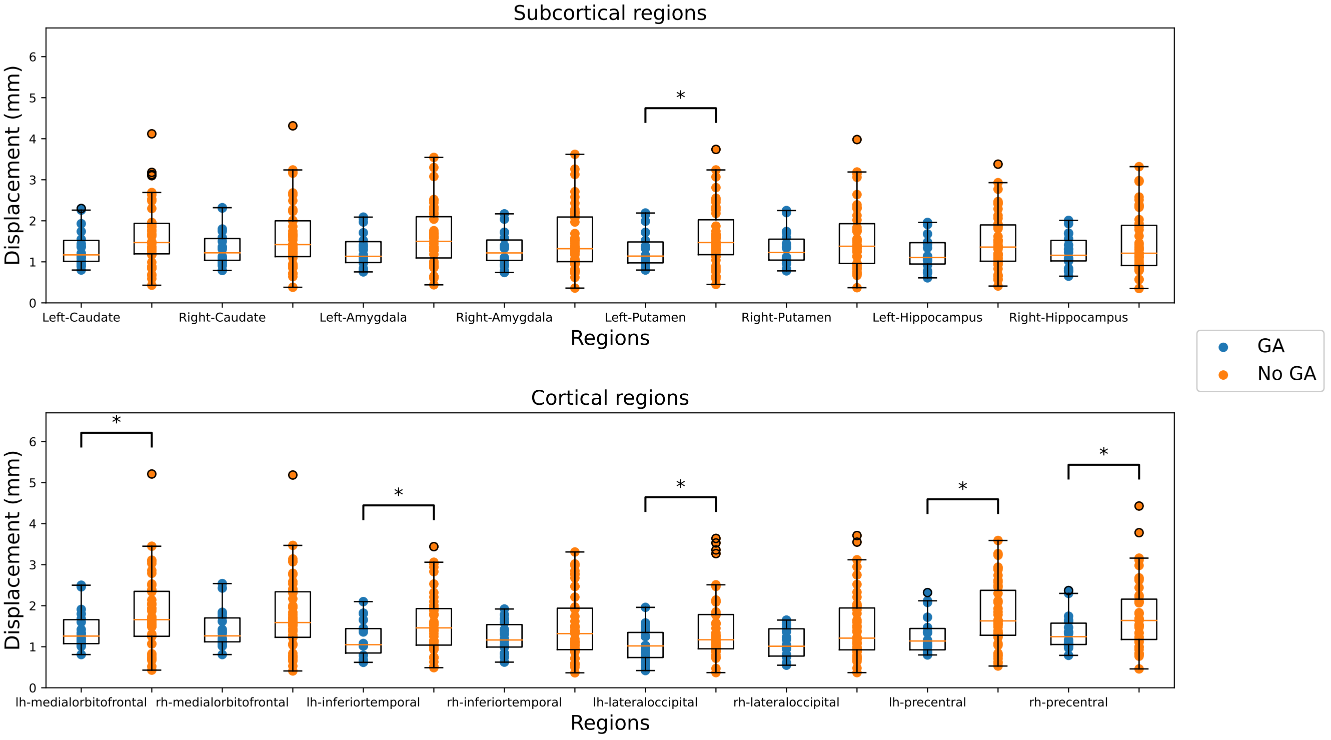

Figure S5. Comparison of median displacement for all cortical and subcortical regions. Statistical significant differences after Benjamini-Hochberg correction are indicated by * ($p < 0.05$).

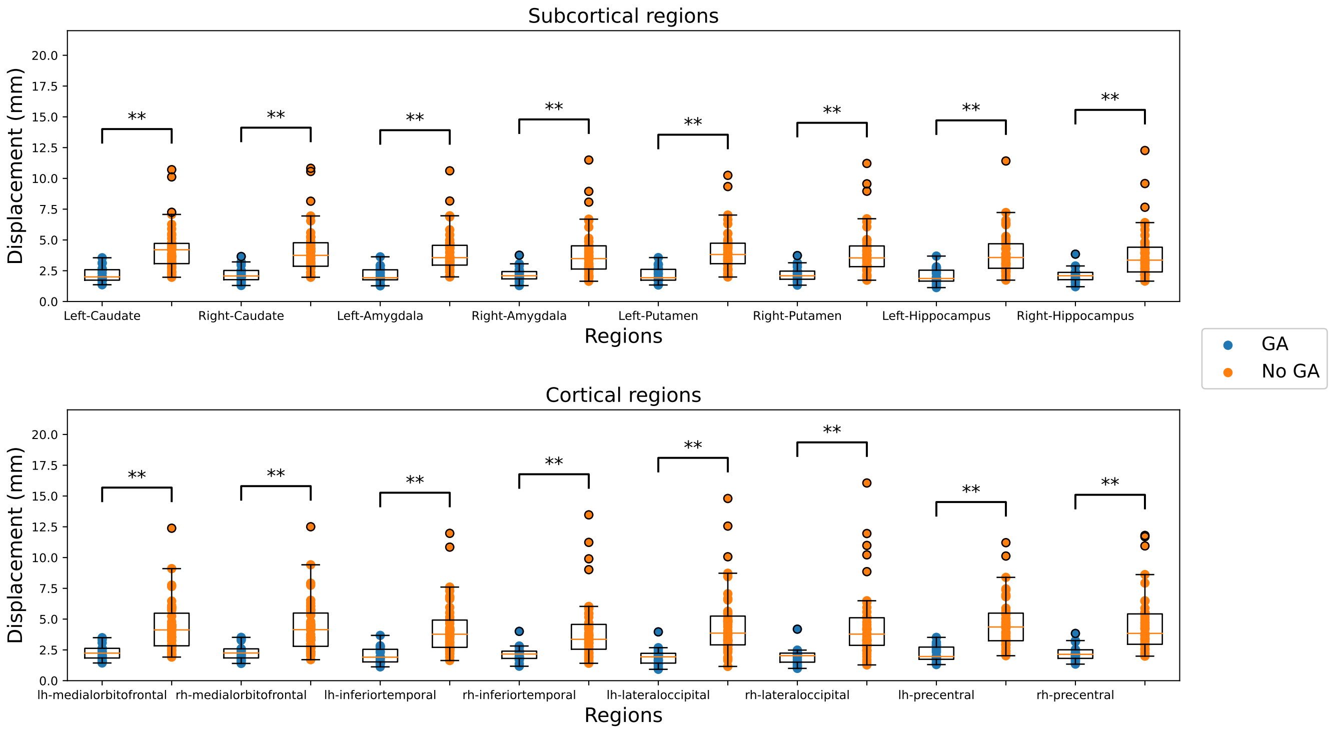

Figure S6. Comparison of maximum displacement for all cortical and subcortical regions. Statistical significance after Benjamini-Hochberg correction is indicated by ** ($p < 0.001$).

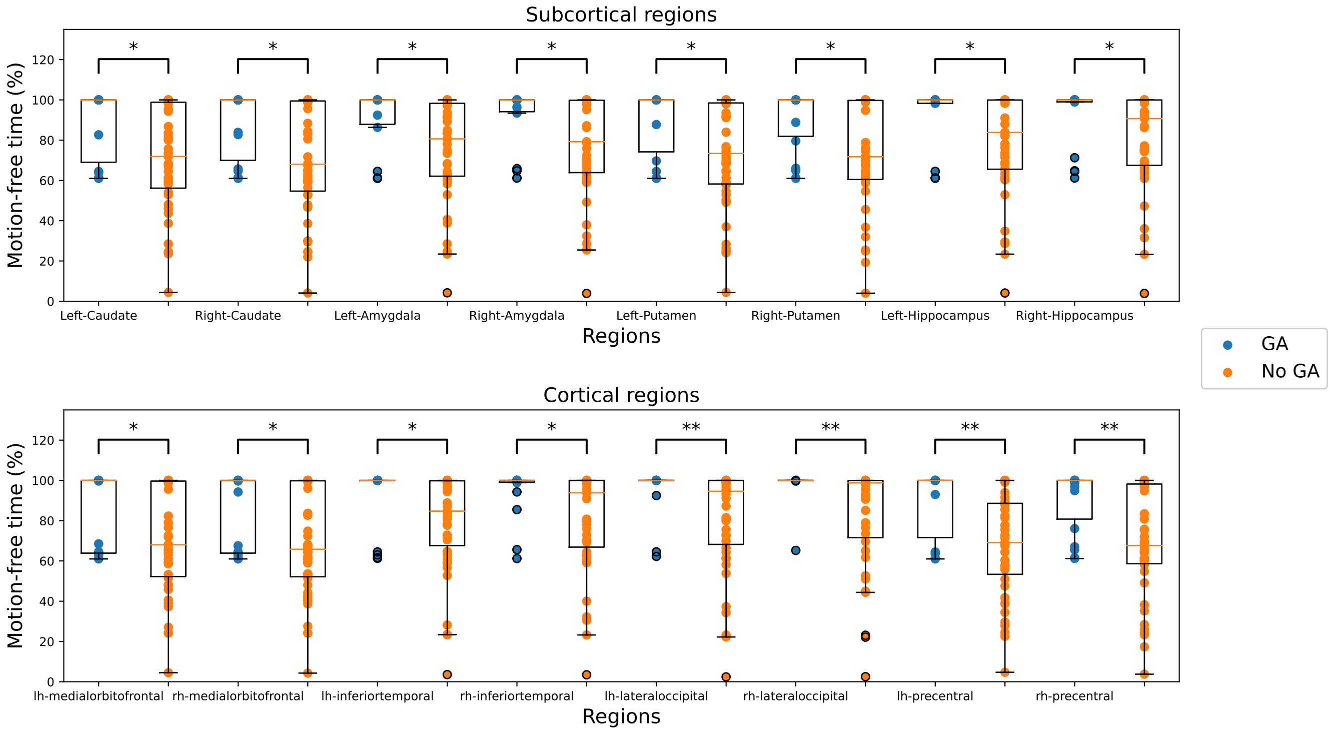

Figure S7. Comparison of motion-free time for all cortical and subcortical regions. Statistical significance after Benjamini-Hochberg correction is indicated by */** ($p < 0.05/p < 0.001$).

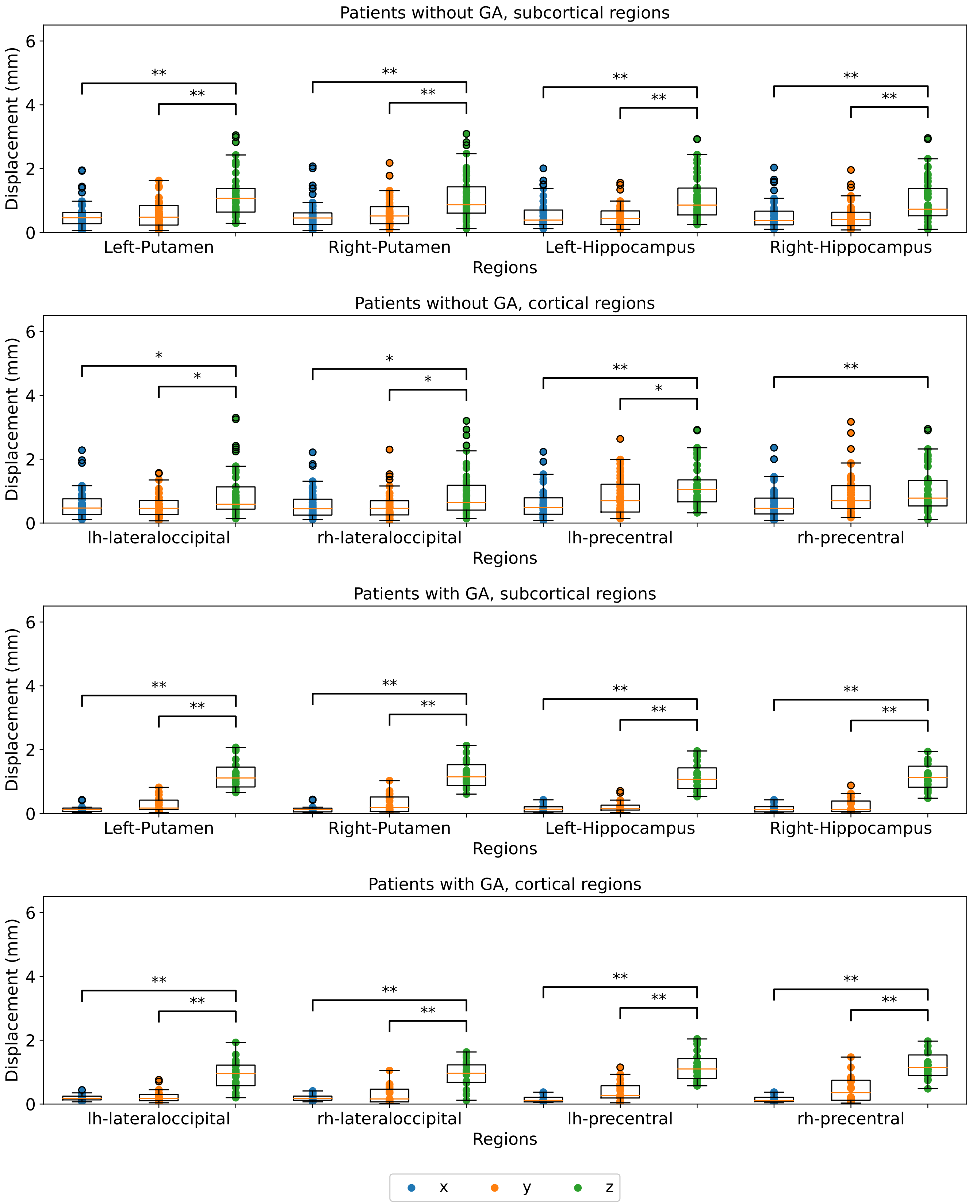

Figure S8. Comparison of median displacement on each axis for the 4 cortical and 4 subcortical regions not included in Figure 6. The top two plots show patients without GA, the lower two patients with GA. Statistical significance after Benjamini-Hochberg correction is indicated by */** ($p < 0.05/p < 0.001$).
